# Podophyllin Poisoning

**Published:** 1890-07-12

**Authors:** 


### PODOPHYLLIN POISONING.

Dr. Dudley, of Newport, N.Y., relates a case of fatal podophyllin poisoning, in a recent number of the "N.Y. *Medical Record*. The quantity taken, by mistake, was five grains of the resinoid podophyllin. The first effects were vomiting and purging of bilious matter, with symptoms of collapse. Twenty-four hours afterwards the patient became comatose. Dr. Dudley remarks that the symptoms suggested that the liver had been stimulated to the production of a large amount of cholic, choleic, taurocholic, or glycocholic acid, the effect of which was to bring about a condition similar to that of cholæmia, minus the jaundice. It is not often that poisoning by podophyllin has occurred, in fact the above case is almost unique. The symptoms, however, as detailed by Dr. Dudley, accord pretty much with the action of podophyllin on animals as described by Dr. Anstie, Dr. Rutherford, and the Edinburgh committee presided over by Dr. Bennett. The British Pharmacopœia gives the dose of resin of podophyllum as from ¼ to 1 grain, but we have rarely prescribed more than the first quantity.

